# Precise Terminology and Specified Catheter Insertion Length in Ultrasound-Guided Infraclavicular Central Vein Catheterization

**DOI:** 10.3390/medicina60010028

**Published:** 2023-12-23

**Authors:** Ainius Žarskus, Dalia Zykutė, Saulius Lukoševičius, Antanas Jankauskas, Darius Trepenaitis, Andrius Macas

**Affiliations:** 1 Department of Anesthesiology, Lithuanian University of Health Sciences, 44307 Kaunas, Lithuania; dalia.zykute@stud.lsmu.lt (D.Z.); darius.trepenaitis@lsmu.lt (D.T.); andrius.macas@lsmu.lt (A.M.); 2 Department of Radiology, Lithuanian University of Health Sciences, 44307 Kaunas, Lithuania; saulius.lukosevicius@lsmu.lt (S.L.); antanas.jankauskas@lsmu.lt (A.J.)

**Keywords:** axillary vein, central venous catheters, subclavian vein, interventional ultrasonography

## Abstract

*Background and Objectives*: As the latest research encourages the ultrasound-guided infraclavicular central venous approach, due to the lateral puncture site displacement, in comparison to the anatomical landmark technique based on subclavian vein catheterization, the need to re-calculate the optimal catheter insertion length and possibly to rename the punctuated vessel emerges. Although naming a particular anatomical structure is a nomenclature issue, a suboptimal catheter position can be associated with multiple life-threatening complications and must be avoided. The main study objective is to determine the optimal catheter insertion length by the most proximal ultrasound-guided, in-plane infraclavicular central vein approach, to compare results with the anatomical landmark technique based on subclavian vein catheterization and to clarify the punctuated anatomical structure. *Materials and Methods*: 109 patients were enrolled in this study. All procedures were performed according to the same catheterization protocol. In order to determine optimal insertion length, chest X-ray scans with an existing catheter were performed. The definition of punctuated vessel was based on computer tomography and evaluated by radiologists. Independent predictors for optimal insertion length were identified, prediction equations were generated. *Results*: The optimal catheter insertion length is approximately 1.5 cm longer than estimated by Pere’s formula and can be accurately calculated based on anthropometric data. Computed tomography revealed: five cases with subclavian vein puncture and three cases with axillary vein puncture. *Conclusions*: Even the most proximal ultrasound-guided infraclavicular central vein access does not guarantee subclavian vein catheterization. A more accurate term could be infraclavicular central venous access, with the implication that the entry point could be through either subclavian or axillary veins. The optimal insertion length is approximately 1.5 cm deeper than the length determined for the anatomical landmark technique based on subclavian vein catheterization.

## 1. Introduction

While our latest research encourages the use of ultrasound-guided infraclavicular central venous access during elective surgery [1], the in-plane approach shifts the puncture site laterally in comparison with the anatomical landmark technique based on subclavian vein catheterization; therefore, the precise vessel’s definition and optimal catheter insertion length must be re-evaluated. Since the boundary separating the subclavian and axillary veins passes through the lateral edge of the first rib and the subclavian vein is mostly located behind the clavicle and, as a result, bone shadowing impairs the vessel’s visualization, an opinion is expressed in the current debate that the vein visible on the ultrasound image in the infraclavicular area is generally axillary vein [2,3]. As a consequence, the ability to reach subclavian vein is technically doubtful [4]. Some authors even state that ultrasound-guided subclavian vein catheterization can be performed only using the supraclavicular approach [5]. Displacement of the puncture site laterally would increase the optimal insertion depth, and Pere‘s formula described in the 1990s for the prediction of the appropriate, anatomical landmark technique based subclavian vein catheter length may not perform well [6]. Too-shallow insertion, as well as inappropriate catheter selection, carries risks of not-parallel catheter tip alignment with the vessel‘s wall, which in turn increases the risk of a vascular injury [7,8] and, subsequently, vein thrombosis [9,10] and infection [11,12]. On the other hand, intracardiac placement may cause heart structure laceration or even heart perforation [13,14,15].

## 2. Materials and Methods

### 2.1. Ethical Statement

This study is a continuation of our previously published research, which was conducted to evaluate the technical challenges, safety and complications of ultrasound-guided infraclavicular central access in elective surgery [1]. The goal of this paper was to analyze the relevant anatomy to determine the precise terminology and optimal catheter insertion length. The approval of the regional biomedical research ethics committee was obtained (No. BE-2-15), and the ethical principles of the Declaration of Helsinki were followed. Every patient was informed and gave their written consent before participating in the study.

### 2.2. Enrollment and Study Protocol

This was a prospective, observational, single-center study carried out in the Hospital of the Lithuanian University of Health Sciences Kaunas Clinics. The study took place from October 2020 until May 2021. In total, 109 patients underwent US-guided infraclavicular central vein catheterization. All catheterizations were performed following an evidence-based protocol by all anesthesiologists. The protocol was developed with an aim of having technical peculiarities similar to the catheterization of the internal jugular vein, which is familiar to operating room staff. In this way, the transition to the new technique for everyone involved would be as effortless as possible. All study participants under general endotracheal anesthesia were positioned in the 15° Trendelenburg position [16,17]. Under strict sterility requirements, the subclavian and axillary veins were visualized in the longitudinal plane. In order to ensure a maximally proximal puncture, the medial edge of the transducer was positioned slightly above the clavicle. The puncture was performed without a syringe, with a guidewire preloaded into the needle. The introducer needle was inserted strictly in the longitudinal plane, with the cut of the needle clearly identified on the ultrasound image. After the needle reached the lumen of the vein, the guide was immediately inserted under direct ultrasound observation. Only after confirming the intravascular position of the guidewire, the dilator followed by the catheter was inserted. Due to the presumed slightly distal approach and in order to ensure an accurate calculation of the optimal catheter insertion depth, all catheters were inserted to a depth of 20 cm. In order to protect patients from harm, catheterization must have been completed within 15 min, and the maximal allowed needle redirections were 2. The catheters were deemed as safe to use if free aspiration was observed from all ports and the tracing obtained from the catheter was venous. Conventional chest radiograms were obtained to confirm the catheter’s position in the post-anesthesia care unit. The ethical committee board permitted us to obtain CT scans of the chest only if necessary; hence, 8 patients had medical indications for chest CT scans, which were evaluated to determine the precise catheterized vessel—either axillary or subclavian veins. All catheters were right-sided. For detailed a protocol of infraclavicular US-guided central vein catheterization, the reader is advised to refer to our previously published paper [1], and a video demonstration at (https://youtu.be/lSsnmeXvIeI) (accessed on 8 November 2023).

### 2.3. Determination of Catheter’s Length, Position and Relevant Anatomy

**Figure 1:**
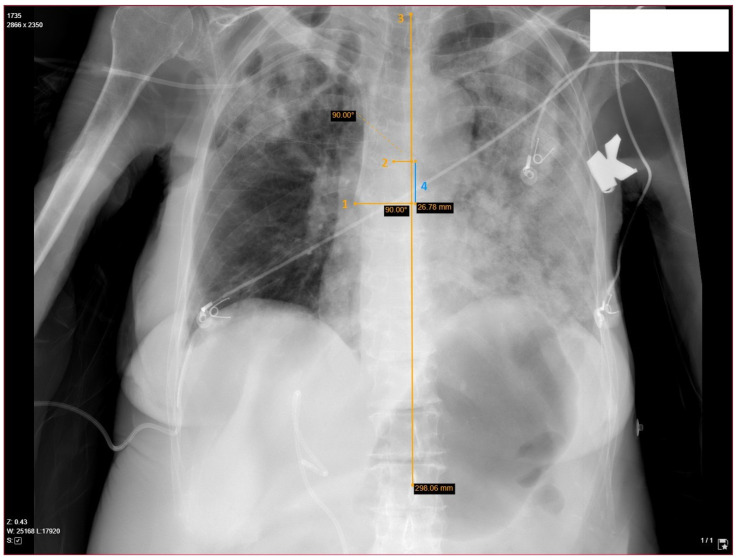
Catheter’s tip confirmation and optimal insertion length measurement demonstrated with yellow markings. Chest X-ray in the supine position obtained after surgery: after determining the patient’s axis using 1st and 12th thoracic vertebrae as landmarks (3), the distance between the carina and catheter’s tip are measured on the axis (4), which is subtracted or added to 20 cm (default insertion length in this study). 1—catheter’s tip, 2—carina, 3—patient’s axial axis, 4—catheter’s tip shift from the ideal depth.

**Figure 2:**
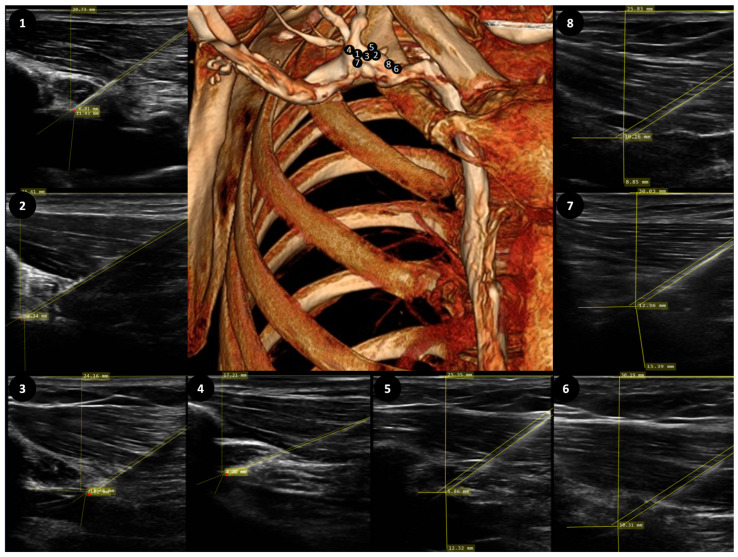
3D CT and ultrasound images with marked puncture locations. According to ultrasound imaging taken during the procedure, the puncture location with respect to the clavicle in both groups did not differ. Numbered ultrasound images, showing entry to the vein, correspond to the number on the reconstructed CT scan, which show the location of the vessel’s puncture. 1, 4, 7 represent entry through the axillary vein; 2, 3, 5, 6, 8—represent entry through the subclavian vein.

Anteroposterior chest X-ray scans were used to determine the ideal catheter’s length. The ideal catheter insertion length was estimated for a particular patient on the MedDream DICOM platform (Figure 1). According to the currently available evidence [18], alignment to the carina level was chosen as an optimal catheter tip position. Measurements were made in a stepwise fashion. First, the patient’s axial axis was determined based on alignment through the 1st and 12th thoracic vertebra. Then, the catheter’s tip and carina were marked and perpendicular lines were drawn to the axial axis. The distance between the lines was used to determine the distance from the ideal position. The optimal insertion length was calculated by retracting or adding this distance to the inserted catheter’s length (20 cm), depending on the catheter’s position (below or above carina). Contrast-enhanced CT scans with 3D reconstructions were obtained and evaluated by two certified radiologists, focusing on the catheter’s entry point to the vessel. The entry point was described with respect to the 1st rib and cephalic vein. Subclavian access was considered if the puncture point was intrathoracic and axillary access was considered if the puncture point was extrathoracic (Figure 2).

### 2.4. Statistical Analysis

The data were analyzed using the SPSS v25.0 (IBM Corp. Released 2017. IBM SPSS Statistics for Windows, Version 25.0. Armonk, NY, USA: IBM Corp.) software. The Kolmogorov–Smirnov test was used to assess data normality. The mean and standard deviation (SD) were used to report normally distributed data, whilst the median and interquartile range (IQR) were used for non-normally distributed data. To identify factors affecting optimal insertion length, linear regression was applied. Paired samples *t*-test and one-sample *t*-tests were used for comparisons.

## 3. Results

### 3.1. Demographic and Anatomical Parameters

In total, 109 cases were enrolled into the study, 2 were eliminated because of a failed procedure, and 9 were eliminated because of misplacement of the catheter’s tip to the right internal jugular vein or left brachiocephalic vein. In total, 49 men and 49 women were included in the final analysis. Age, weight, height, vein entry distance from the clavicle, and ideal catheter insertion length fulfilled normal distribution criteria. The ideal catheter insertion length on average was 168.03 mm (SD 18.0 mm) and varied from 128.3 mm to 216.8 mm (Table 1). A total of 9 cases were evaluated with a contrast-enhanced CT scan. There was a significant increase in the optimal insertion length by 14.3 mm (SD 8.99 mm, *p* < 0.001) when compared with the length calculated using Pere’s formula.

### 3.2. Predictors of Optimal Catheter Insertion Length

**Figure 3:**
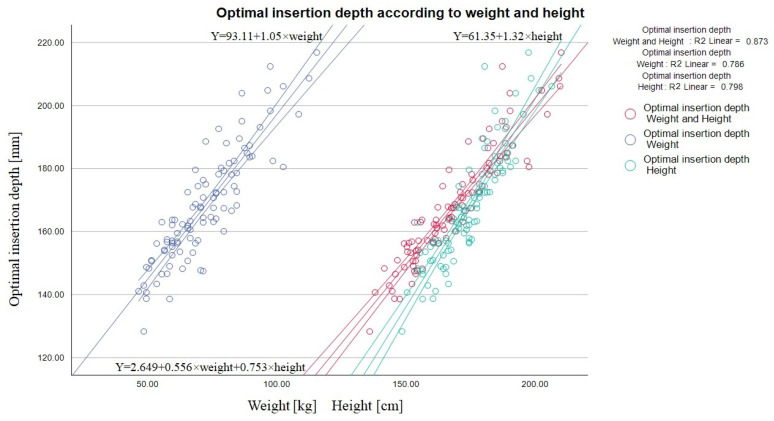
Linear regression graph showing dependency of the skin–carina distance [mm] and patient’s weight [kg] R^2^ = 0.786, height [cm] R^2^ = 0.798, and in conjunction R^2^ = 0.873. The patient’s weight and height significantly influences the catheter’s predicted optimal length and hence could be used to determine the optimal catheter based on the formula generated (optimal catheter insertion length [mm] = −2.649 + 0.556 × (weight [kg]) + 0.753 × (height [cm]).

Multivariable linear regression model analysis showed the ideal catheter’s length moderate dependency on the vein’s depth, diameter, puncture angle, and path length (R—0.580) (Table 2). Prediction equation: optimal insertion length [mm] = 209.822 + 1.646 × (vein diameter [mm]) + 5.571 × (vein depth [mm]) − 2.056 × (puncture path [mm]) − 2.925 × (puncture angle [degrees]). Since these factors are influenced the by patient’s anthropometric qualities, such general parameters as weight and height show a better prognostic value in coefficients of determination (R^2^) of weight—0.786—and height—0.796 (Figure 3).

Combining these parameters together provides an even higher prognostic benefit R—0.934 (R^2^—0.873) and in 87% of cases correctly predicts optimal insertion length (R—0.934, Durbin–Watson—1.465). Prediction equation: optimal catheter insertion length [mm] = −2.649 + 0.556 × (weight [kg]) + 0.753 × (height [cm]) (Table 2) (Figure 3).

### 3.3. Precise Terminology

In total, 8 contrast-enhanced chest CT scans were evaluated. In 5 cases, entry was in the subclavian vein as determined by the puncture point medial to the lateral boarder of the 1st rib. In 3 cases, entry was in the axillary vein as the puncture point was lateral to the border of 1st rib (Figure 2). Ultrasound imaging parameters, such as distance from the vein entry point to the clavicle shadow, did not reach statistical significance (Table 3).

## 4. Discussion

Damage to vital structures and serious complications can arise not only because of the puncture technique, but also from secondary displacement of the catheter due to movement of the arm, torso or even the blood vessel itself. Life-threatening arrhythmias [19], tricuspidal valve damage or even heart perforation are described in the literature [13,14,15].

The optimal catheter tip position still remains controversial. In 1989, the US Food and Drug Administration stated: “central venous catheter should not be placed in, or allowed to migrate into the heart” [20]. Logically, at that time great attention was paid to avoid too-deep catheter placement. Later on, evidence revealed the importance of the catheter tip and vessel wall alignment—angles above 40 degrees should be avoided as this carries a higher rate of vessel wall erosion [7]. Since the left brachiocephalic vein joins the superior vena cava at a steeper angle, a left-sided catheter’s tip usually takes a parallel orientation deep enough, mostly below the carina. This problem is less pronounced with right-sided catheters: merely 2.4% of inserted catheters (4/163) have an angle steeper than 40°, compared to 63% (27/43) of left-sided catheters. Therefore, right-sided catheters can also be safely secured in the vena cava—above the carina level—and do not necessitate such a deep insertion [7,8]. For this reason, the separation of the vena cava superior into two different zones was necessary.

However, lately, an up to 16-times-higher risk of thrombotic complications by insufficient catheter insertion depth has been revealed. In total, 41.7% of the patients with long-term catheters placed in the proximal third of the vena cava developed thrombotic events, compared to 2.6% of patients with a catheter placed in the distal third of the vessel [9,10]. In addition to flow obstruction and thromboembolic events, thrombosis is also associated with catheter-related sepsis [11,12]. The better antimicrobial properties of deeper inserted catheters have been demonstrated [21]. Furthermore, randomized trial with large-bore dialysis catheters located in the right atrium (81%) failed to prove an increase in arrhythmic or mechanical complications [22], which raises doubts about the validity of the claims made by the US Food and Drug Administration in 1989.

Moreover, a deep inserted central venous catheter has one important positive practical aspect—the catheter can always be withdrawn without any distal contamination. However, central venous catheters which are not inserted deep enough would require replacement [3].

Various catheter tip confirmation methods are established. Atrial electrocardiography [23,24], and echocardiography [25] have been described; however, we adopted an ordinary chest radiogram, which also allowed calculations of optimal catheter insertion length and the ability to compare with already published results. However, the identification of the pericardium in an ordinary chest radiogram is aggravated; therefore, extracardiac tip placement is difficult to establish. Several different radiological landmarks to identify the desired location are already described [18,26]. Research with fresh cadavers disclosed the carina’s location from 0.05 cm to 0.1 cm above the pericardial sac [18,27] and can be used as a landmark to determine the optimal position of the catheter’s tip. Similar results were found in a different study with live subjects, where CT scans revealed that even though the carina appeared 0.7 cm below the pericardial reflection, it was still above the entrance to the atrium by 2.9 cm [26]. The results from open cardiac surgery are almost identical—in 95% cases, the carina was situated 2.5–6.2 cm above the superior vena cava and atrium junction [28].

In 1995, Peres generated a formula for right subclavian catheter insertion length − height/10 − 2 which in 97% of cases correctly predicts superior vena cava tip placement [6]. As expected, in our study, the optimal insertion length significantly exceeded optimal length calculated by Pere’s formula—mean difference 14.3 mm (SD 8.99 mm). The actual length was found to be 168 mm (SD 18.03 mm) compared to the predicted 153 mm (SD 12.2 mm, *p* < 0.005). The result confirms that even the most medial infraclavicular ultrasound-guided access, which was 10.30 mm (SD 4.0 mm) from the acoustic shadow of the clavicle, will have a significant lateral shift in comparison with anatomical landmark access, and will increase the optimal catheter insertion depth. In our study, the predicted length was also higher than the one reported in a study by Won Young Kim and colleagues. They reported an optimal length of 14 cm when using the anatomical landmark catheterization technique with the mean difference from our study being 28 mm (one sample *t*-test, *p* < 0.001) [29]. The increase in length in our study can be attributed to a more lateral punctuation point due to the usage of ultrasound guidance and, perhaps, different anthropological parameters of the populations. Interestingly, a similar study in the Korean population with ultrasound-guided catheterization and fluoroscopic-enhanced carina tip positioning showed the same optimal length of placement as in a previous study (14.10 cm SD 1.46 cm) [30].

Our results are similar to those published by Hyun-Jung Shin and colleagues. In their population, catheter’s length using US-guided insertion technique was 15.4 cm (SD 1.5 cm) [31], slightly lower than the one we report. This could be explained by lower weight and height of the participants in the mentioned study (weight 64.5 kg and height 162.1 cm in comparison to our study’s 71.6 kg and 173.7 cm). When using our proposed formula and adjusting optimal insertion length on the anthropological parameters of mentioned research sample—the result becomes identical (−2.649 + 0.556 × (64.5 [kg]) + 0.753 × (162.1 [cm]) = 15.5 cm).

Our determined optimal depth of catheter insertion is not only greater than the anatomical landmark technique based on subclavian vein catheterization but also varies within very wide limits from 12.8 cm to 21.6 cm depending on the anthropological patient‘s parameters. In our sample, catheter introduction of 20 cm causes a high incidence of malposition—only in 23.4% of cases was the catheter tip found 2 cm above or below the bifurcation of the trachea. Moreover, in 60.2% of cases, the catheter was found more than 2.9 cm below the tracheal bifurcation, which is the mean distance from the carina to the right atrium based on a CT scan of living subjects [26]. A fixed catheter insertion depth at an average value of 16.8 cm still leads to 3% intracardiac placements and 15.3% insufficient depth defined as more than 2 cm above the bifurcation. Such high variability of the optimal catheter insertion depth demonstrates the importance of a method defining the correct catheter tip position. Therefore, the formula generated in our study can be a useful tool for predicting the approximate depth of catheter insertion when intraoperative fluoroscopy or intracardiac electrocardiography is not available.

These findings are also important for choosing the correct catheter length before the procedure, as the widely used 15 cm catheters in many cases will be too short, whereas a 20 cm-long catheter in our sample is suitable for 93.8% of patients. Individuals weighing more than 100 kg and taller than 195 cm mostly require an even longer catheter. Since our previous study revealed the failure of the method in obese patients with a cut-off value of 119.5 kg, the need for a catheter longer than 20 cm in the case of a right subclavicular approach should not be common. However, when the left subclavicular region is selected, assuming that the required insertion depth is 6 cm deeper (left subclavian vein − (height/10) + 4) [6], a 20 cm catheter usually will be insufficient. Additional studies are required to address these issues, so that complications associated with improper catheter placement length can be minimized and patient safety guaranteed in diverse patient populations.

An analysis of CT scans revealed that even the most proximal catheterization technique does not guarantee subclavian access. In our study, in three out of eight cases a vein penetration site was found distal to the lateral edge of the first rib. Thus, naming this catheterization technique “infraclavicular ultrasound-guided central vein access” would be more accurate, with the implication that the entry point could be in the axillary or subclavian vein. While this may only appear as a nomenclature issue, we felt it was important to bring some clarity and ease of communication. The absolute majority of colleagues identify ultrasound-guided central vein access in the subclavicular region as the subclavian vein, although the recorded puncture location is usually even more lateral, without a visible clavicle. Also, we cannot agree with the categorical statement that ultrasound-guided subclavian vein catheterization is only possible with the supraclavicular approach and that the punctured vein in the subclavicular region is exclusively the axillary vein; therefore, we suggest not to mislead each other and to abandon the specific anatomical naming of the vein. Non-specific terms such as the infraclavicular or subclavicular ultrasound-guided central vein approach would be much more appropriate. In addition, correct nomenclature was also important for updating the hospital’s internal central venous catheterization protocol.

### Limitations

Radiographic images are subjected to the parallax effect, which describes the augmentation of structures distant from center of the image. This phenomenon is valid not only for the transversal plane but also the sagittal. On portable X-ray machines, which were used in our study to obtain chest radiographs, this effect tends to be even more pronounced [32]. However, since the carina and superior vena cava are situated almost in the middle of the body, inaccuracies calculating the catheter’s length should be minimized. We were unable to collect a control group of the anatomical landmarks based on subclavian vein catheterization, because there are only a few of our colleagues left practicing this method; the absolute majority perform catheterization of the internal jugular vein under ultrasound control and categorically refused to participate in the study if the procedure would be based on anatomical landmarks. Thus, we were unable to determine our own optimal catheter insertion depth for anatomic landmark-guided subclavian vein catheterization. Therefore, we chose the universally recognized Perez formula to calculate the required catheter insertion depth in this group. The formula is not optimal for our case because it was designed to avoid intra-atrial catheter introduction rather than catheter tip alignment with the carina. We expected to perform more CT scans to characterize the catheter position, but the Bioethics Commission did not allow such a scan for research purposes. The only solution was to wait for medical indications for chest CT scans, which are not common after major abdominal surgeries. Our refined formula for optimal catheter insertion length = 0.556 × weight + 0.753 × height − 2.649 is inconvenient for practical use and should be mathematically simplified. Despite that, it is very important to still estimate the catheter’s optimal length for an individual patient as it can vary widely—from 12.8 cm to 21.6 cm in our study.

## 5. Conclusions

The most proximal ultrasound-guided central vein catheterization does not guarantee access through the subclavian vein; therefore, specific anatomical naming of the vein should be abandoned. Entitlement as infraclavicular central venous access is more appropriate, with the implication that the entry point could be through either subclavian or axillary veins.

The optimal catheter insertion length varies widely and a tool for determining the correct position of the catheter is essential to avoid serious complications such as damage to the vessel’s wall, increased risk of thrombosis, infection or even heart perforation.

Ultrasound-guided central vein access in the subclavicular area leads to an approximately 1.5 cm greater optimal insertion length than the one determined for the anatomical landmark technique by Pere’s formula. Weight and height are reliable predictors of the optimal insertion length and can be adopted to daily clinical practice.

## Figures and Tables

**Table 1:** Demographic and anatomical parameters of patients enrolled into the research. Weight is expressed in kilograms, height in meters, and distances in millimeters.

	Kolmogorov–Smirnov Test of Normality	Mean	Std. Error	Std. Dev
Age	0.200	61.33 years	1.76	17.46
Weight	0.153	71.65 kg	1.54	15.29
Height	0.200	173.71 cm	1.23	12.20
Ideal catheter insertion length	0.73	168.03 mm	1.82	18.03
Pere’s predicted insertion length for the right subclavian vein	0.200	153.7 mm	1.23	12.20

**Table 2:** Factors affecting the optimal catheter’s insertion length. Weight and height are significant independent predictors for deeper catheter insertion.

Optimal Insertion Length	R	Unstandardized B	Coefficient Std. Error	Standardized Coefficients Beta	Sig.
Constant	0.580	209.822	35.383	-	<0.001
Vein diameter		1.646	0.562	0.272	0.004
Vein depth		5.571	1.822	1.687	0.003
Punction path		2.056	0.930	−0.892	0.029
Punction angle		2.925	1.117	−0.997	0.010
Constant	0.934	−2.649	12.247	-	0.829
Weight		0.556	0.074	0.471	<0.001
Height		0.753	0.093	0.510	<0.001

**Table 3:** Distances from the vein entry point to the clavicle shadow for subclavian and axillary access. There was no statistical significance in the difference of the distance from the vein’s puncture point to the clavicle’s acoustic shadow for the subclavian or axillary entry point.

Distance from Vein Entry Point to Clavicle Shadow	Mean (mm)	Std. Error (mm)	Std. Dev (mm)	Median (mm)	Interquartile Range
Axillary vein	6.86	3.18	5.52	6.91	0.0
Subclavian vein	8.54	1.57	3.51	10.16	15.29

## Data Availability

Data can be made available on request from the corresponding author. The data are not publicly available due to patient privacy.
